# The Alteration of Brain Function by the Improvement of Periodontal Tissues and Occlusal State

**DOI:** 10.1155/2022/5383893

**Published:** 2022-04-27

**Authors:** Kosuke Muraoka, Masafumi Oda, Kenichi Yoshino, Tatsurou Tanaka, Masaki Morishita, Taiji Nakamura, Ryota Kibune, Jyunpei Tokunaga, Kazuo Sonoki, Yasuhiro Morimoto, Keisuke Nakashima, Shuji Awano

**Affiliations:** ^1^Science of Oral Functions Department, Division of Clinical Education Development and Research, Faculty of Dentistry, Kyushu Dental University, Japan; ^2^Division of Oral and Maxillofacial Radiology, Kyushu Dental University, Japan; ^3^Section of Primary Dental Education, Kyushu Dental University, Japan; ^4^Graduate School of Medical and Dental Sciences, Department of Maxillofacial Radiology, Kagoshima University, Japan; ^5^Science of Oral Functions Department, Cariology and Periodontology, Division of Periodontology, Kyushu Dental University, Japan; ^6^Unit of Interdisciplinary Education, School of Oral Health Science, Kyushu Dental University, Japan

## Abstract

**Objective:**

In this study, we have introduced a case in which the effective blood oxygenation level-dependent signal on functional magnetic resonance imaging (fMRI) was altered by the improvement of periodontal tissue and occlusal function in a patient with periodontitis Stage II Grade B. *Material and Methods*. A 61-year-old female patient requiring periodontal treatment was diagnosed as having periodontitis Stage II Grade B via clinical and radiographic examinations. Her past medical history included type 2 diabetes, hypertension, and hyperlipidemia. Following the patient's informed written consent, the periodontal initial treatment provided to the patient included tooth brushing instruction and scaling and root planing; however, occlusal adjustment was not performed at this stage. Occlusal force and fMRI results were also evaluated at the initial and reevaluation examinations.

**Results:**

After the periodontal initial treatment had been performed, it was noted that the patient's periodontal tissue and occlusal force had improved. It was also evident from fMRI that cerebral blood flow had been activated in the insula, primary motor cortex, and premotor cortex.

**Conclusion:**

This result suggested that the periodontal ligament had recovered and the periodontal ligament neuron had been further subjected to clenching in the insula so that the muscle spindle sensation impacted the motor cortex.

## 1. Introduction

The relationship between periodontal disease and systemic disease has also been observed in earlier studies. In particular, Nobel et al. [1] noted that periodontal disease caused functional changes in both memory and cognition. In recent years, the relationship between periodontal disease and Alzheimer's disease has been described in studies involving laboratory mice and it was noted that periodontal disease did impact upon brain function [2]. The purpose of periodontal therapy involves improving the function of the tissue as well as improving occlusion function. Therefore, we hypothesized that the improvement of periodontal tissues and occlusal states should alter the effective alteration of brain function. Functional magnetic resonance imaging (fMRI) is one method for evaluating brain activity, and the analysis of the task dominant region of the brain at the central level using fMRI is both a noninvasive and simple procedure.

In this case study, the subject performed clenching before and after periodontal treatment, and using fMRI, it became evident that periodontal treatment contributed to higher brain function.

## 2. Patients

Informed consent was obtained from a patient for being included in the present case (no. 11-39).

A 61-year-old female patient requiring periodontal treatment was diagnosed as having periodontitis Stage III Grade B via clinical and radiographic examinations at the Kyushu Dental University, with a chief complaint of gum pain at the mandible molar. Her history of dental present illness was diagnosed with periodontal disease after dental examination several months ago. Her medical history was hypertension, type II diabetes, and hyperlipidemia, and she took angiotensin II receptor antagonist (20 mg/per day), biguanide oral hypoglycemic agent (250 mg/per), and MG-CoA reductase inhibitor (5 mg/per day). The patient's physical condition was moderate, with no abnormalities evident. Her dental history had some previous dental treatment done; however, this did not include any periodontal treatment. Her family history and habits had nothing of relevance.

At first visit and reevaluation, periodontal clinical parameters such as probing pocket depth (PPD), clinical attachment level (CAL), bleeding on probing as a percentage of the measured area (%BOP(+)), and tooth mobility were examined. PPD and CAL measurements of the mesial, central, and distal of the buccal and lingual side of each tooth were achieved using William's probe. %BOP(+) occurred within 30 seconds after probing. The Miller [3] classification of tooth mobility was used to categorize from 0 to 3 degrees.

Following the patient's informed written consent, the periodontal initial treatment provided to the patient included tooth brushing instruction and scaling and root planing under local anaesthesia. Furthermore, ultrasonic cleaning with tap water was performed after every treatment. However, occlusal adjustment was not performed at this stage.

The subject was made level with the camper plane and the floor in the sitting position, and a pressure-sensitive measurement sheet (Dental Prescale® 50H-R type, GC CO., Tokyo) was inserted into the mouth. At this time, the subject chewed at the occlusal engagement position with the maximum occlusal force for 3 seconds and the occlusion state was recorded. This procedure was then repeated three times.

The MRI apparatus used (1.5 T full-body MR system, EXCELART Vantage™ Powered by Atlas; Toshiba, Tokyo, Japan) was provided by the Dental Radiology Department of the Kyushu Dental University. Observance of the brain activity site using the fMRI measurement was used as a method of Oda et al. [4]. The subject entered the MRI room and was provided with earplugs to protect their ears from noise during the MRI process. They were then photographed in a supine position with the eye ear plane set so as to be perpendicular to the floor surface. In addition, to prevent the head from moving, the forehead was held in position with a pad and belt. The subject was given instructions from the control room and was asked to carry out a chewing motion on both sides of her mouth. The task of double-sided bite occlusion for 30 seconds and nonocclusion for 30 seconds was repeated three times. Data analysis was performed using SPM 8 (http://www.fil.ion.ucl.ac.uk/spm/software/spm8) executed by Matlab 7.11 (MathWorks, Sherborn, MA, USA). The images from the functional scans were normalized to the standard Montreal Neurological Institute template. The *t*-test was used to determine the significance of the voxels. Activation areas were characterized by their peak heights (*p* < 0.01).

## 3. Results

At the initial visit, gingival swelling and redness of the periodontal tissue were evident; however, these symptoms had improved by the time of reevaluation.

Periodontal tissue examination was indicated (Table 1). The average PPD at the initial visit was 3.3 mm, and the average PPD at the reevaluation was 2.3 mm. The average CAL at the initial visit was 3.4 mm, and the average CAL at the reevaluation was 2.5 mm. The average %BOP(+) at the initial visit was 69.6%, and the average %BOP(+) at the reevaluation was 21.7%. The average tooth mobility at the initial visit was 0.2, and the average mobility at the reevaluation was 0.0 using the Miller [3] classification method.

**Figure 1:**
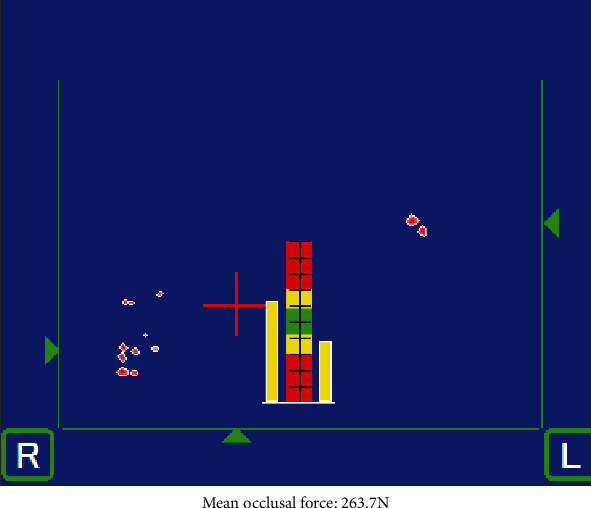
Occlusion state at initial visit. Mean occlusal force: 263.7 N.

**Figure 2:**
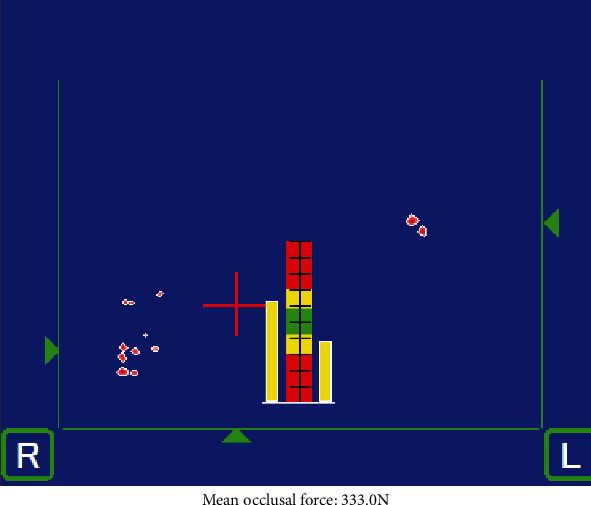
Occlusion state at reevaluation visit. Mean occlusal force: 333.0 N.

The occlusal force was 263.7 N at the initial visit. In the occlusion state at the first visit, occlusal contact was not observed for the anterior tooth, but rather occlusal contact was made at the right molar tooth. Occlusal balance slightly favored the left side (Figure 1). The occlusal force was 333.0 N at the reevaluation. In the occlusion state at the reevaluation, occlusal contact was once again not observed in the anterior tooth, but was made at the right molar tooth. Occlusal balance was the same as that of the initial visit (Figure 2).

**Figure 3:**
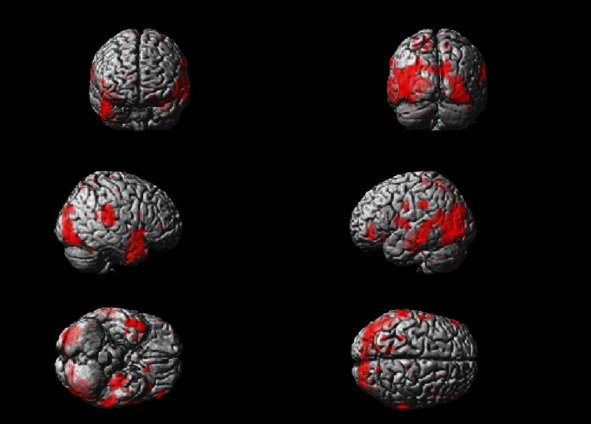
Brain activation image of clenching task at initial visit.

**Figure 4:**
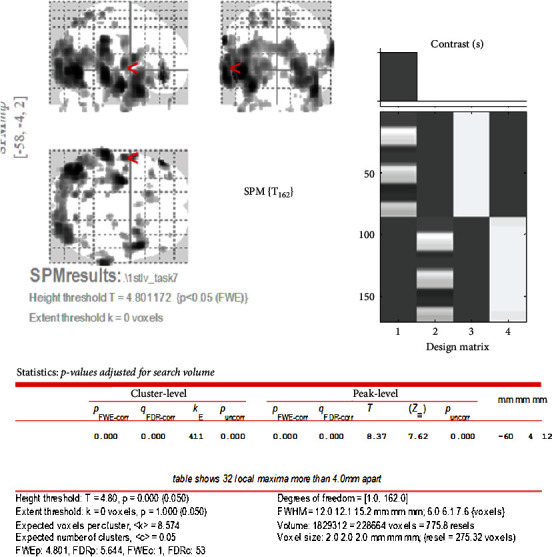
*T* value of brain activation of clenching task at initial visit.

**Figure 5:**
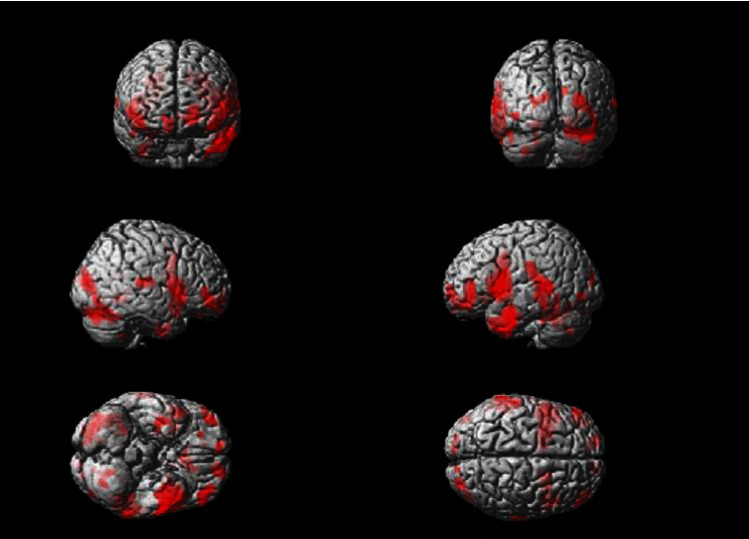
Brain activation image of clenching task at reevaluation visit.

**Figure 6:**
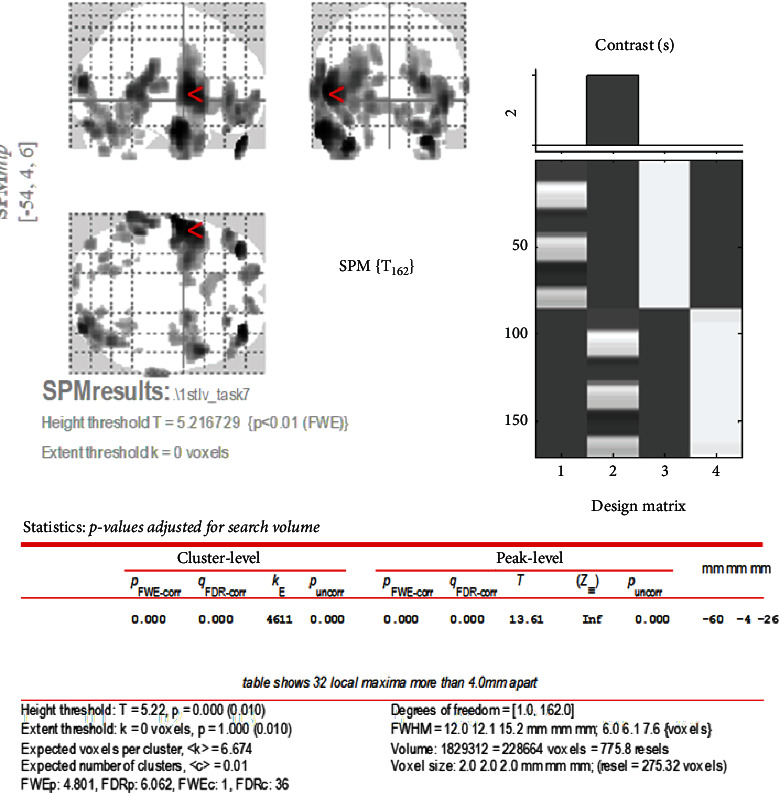
*T* value of brain activation of clenching task at reevaluation visit.

Brain activity from the initial visit occurs in the motor area of the insula, the primary motor cortex, the primary somatosensory cortex, and so on (Figure 3). The *T* value was recorded as 8.37 as can be seen in Figure 4. Increased brain activity from the reevaluation occurs in the same area as that of the first visit (Figure 5). However, the *T* value in the insula was significantly higher than that of initial visit. The *T* value was recorded as 13.61 as can be seen in Figure 6.

The nature of periodontal disease was explained to the subject at the initial visit, and the treatment was set as tooth brushing instruction by bass method, scaling, root planing, and the use of an ultrasonic scaler for each visit. The periodontal initial treatment period was roughly 5 months.

## 4. Discussion

Periodontal treatment is aimed at eliminating the cause of periodontal disease, stopping the progress of lesions, and eliminating factors such as plaque and calculus from occurring. Previous clinical evaluations [5] had largely focused on PPD, CAL, and radiographs; however, there were very few reports that targeted the functional changes of occlusion [6]. Makino et al. [6] concluded that occlusal force increases with periodontal treatment and that the amount of change was seen to be significantly larger in the molars than in the anterior teeth and that inflammatory changes in periodontal tissue and occlusal force are closely related. In this case, the improvement of periodontal tissue and an increase of occlusal force were observed.

We assumed that the occlusion state was controlled at the central level of local movement and perception and that by using fMRI we could see changes in the central nerve level control due to changes in periodontal tissue before and after periodontal treatment.

Belliveau et al. [7] were the first to perform fMRI. fMRI is a technique that captures the slight signal enhancement of blood oxygenation in blood vessels caused by increased neural activity. The fMRI technique uses the blood oxygenation level-dependent (BOLD) effect [8] to detect changes in the balance between regional cerebral blood flow and oxygen metabolism and then reconstructs them in functional images. Because the fMRI is noninvasive, has excellent temporal and spatial resolution, and does not involve radiation exposure, it has been used in a variety of settings. To date, there have been few reports conducted on the relationship between periodontal treatment and the brain using fMRI, so in this case study, our objective was to investigate this particular relationship.

Results from fMRI at the initial visit showed that in the state of biting cerebral activity was evident in the insula. These results demonstrated that occlusal contact was larger on the left side than that of the right and that periodontal ligament perception only reacts on the left side at the central level. The occlusion status of the subject showed not only the task level but also the left biting at the central level. In the reevaluation after initial periodontal treatment and in the state of biting, increased brain activity was observed in the motor area of the insula, when compared to the initial examination. In addition, new activation was found in the ventral part, including the primary motor cortex and premotor cortex. It also demonstrated an increase in occlusal force at the central level.

The insular cortex is involved in tongue and jaw movements, and the ventral part of the insular cortex, including the primary motor cortex and premotor cortex, is involved in the expression of voluntary movements. It was reported that electrical stimulation of the insular in rats induced rhythmic jaw movements similar to masticatory movements and active salivation [9]. In addition, Sörös et al. [10] reported that the insular was activated during water swallowing in humans. When swallowing is accompanied by jaw movement, we assumed that the insular is activated as reported by Maeda et al. [10].

The *T* value is a t-map that shows the test statistic calculated for each voxel as a result of the *t*-test performed on each voxel and illustrated on the brain image template. This means that the spatial specificity is higher and the brain activation can be localized. In this case study, we could identify the brain activation site from the *T* value at the initial examination (Figure 5) and the *T* value at the reevaluation (Figure 6).

These results suggest that periodontal treatment increases bite force and jaw movement by improving the periodontal tissue and thus leading to activation of the insular of the brain.

Butera et al. [11–13] reported significant improvements in periodontal tissue with the use of ozonized water and chlorhexidine. The use of these substances for periodontal tissue and brain activation is an issue for further investigation.

In conclusion, this case is but one and the findings are assumed. We believe the change in the BOLD signal was not only a change in occlusal state but that another factor is also highly involved. Therefore, we plan to increase the number of case studies and continue to research and scrutinize our findings in the future.

## 5. Conclusion

In this study, we reported an interesting case in which improvement of occlusal function in a chronic periodontitis patient was able to be confirmed by the BOLD signal on functional magnetic resonance imaging.

These fMRI results suggested that the occlusal condition had changed from a weak bite on only one side to a strong bite on both sides following the initial periodontal treatment regimen.

## Figures and Tables

**Table 1:** Comparison of periodontal tissue and occlusal force at the initial visit and reevaluation.

	Initial visit	Reevaluation
PPD (mm)	3.3	2.3
CAL (mm)	3.4	2.5
%BOP(+)	69.6	21.7
Mobility	0.2	0.0
Ave occlusal force (N)	263.7	333.0

## Supplementary Materials

Supplementary Materials STARD 2015 checklist and CARE checklist.
