# Supplementary figures and images for: The Alteration of Brain Function by the Improvement of Periodontal Tissues and Occlusal State
Source: Case Rep Dent. 2022 Apr 27;2022:5383893. doi: 10.1155/2022/5383893 (PMC9068291; doi:10.1155/2022/5383893)

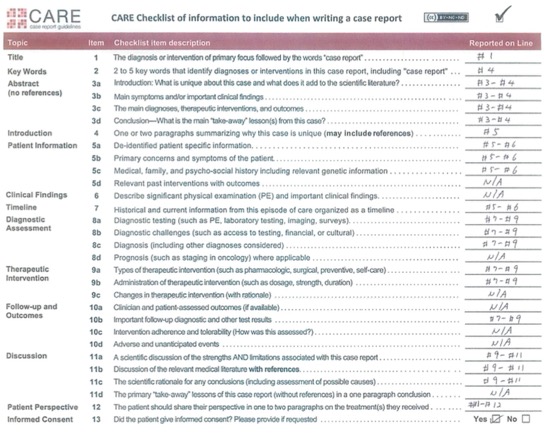

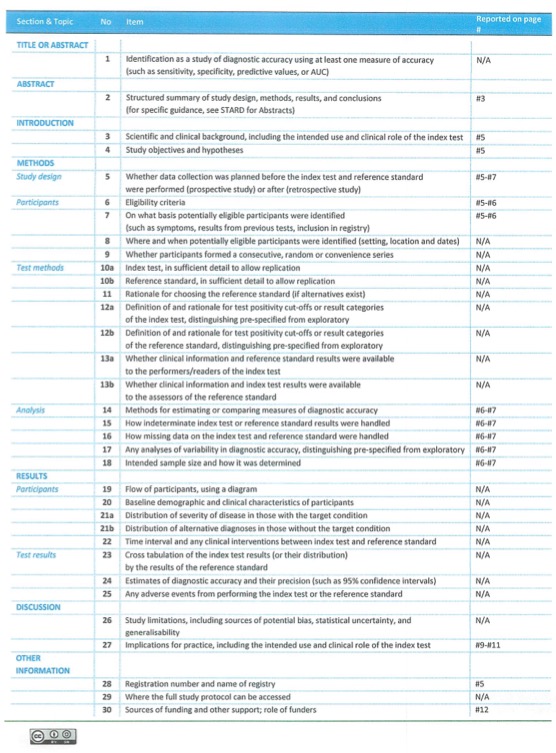

Supplement: Supplementary Materials — STARD 2015 checklist and CARE checklist. [file 5383893.f1.docx]
